# Histone Modifications of H3K4me3, H3K9me3 and Lineage Gene Expressions in Chimeric Mouse Embryo 

**DOI:** 10.22074/cellj.2020.6443

**Published:** 2019-09-08

**Authors:** Maryam Salimi, Abolfazl Shirazi, Mohsen Norouzian, Mohammad Mehdi Mehrazar, Mohammad Mehdi Naderi, Mohammad Ali Shokrgozar, Mirdavood Omrani, Seyed Mahmoud Hashemi

**Affiliations:** 1 Department of Biology and Anatomical Sciences, Faculty of Medicine, Shahid Beheshti University of Medical Sciences, Tehran, Iran; 2 Reproductive Biotechnology Research Center, Avicenna Research Institute, ACECR, Tehran, Iran; 3 Department of Gametes and Cloning, Research Institute of Animal Embryo Technology, Shahrekord University, Shahrekord, Iran; 4 National Cell Bank of Iran, Pasteur Institute of Iran, Tehran, Iran; 5 Department of Medical Genetics, Shahid Beheshti University of Medical Sciences, Tehran, Iran; 6 Department of Immunology, School of Medicine, Shahid Beheshti University of Medical Sciences, Tehran, Iran

**Keywords:** Cell Lineage Genes, Chimera, H3Methylation

## Abstract

**Objective:**

Chimeric animal exhibits less viability and more fetal and placental abnormalities than normal animal. This study was aimed to determine the impact of mouse embryonic stem cells (mESCs) injection into the mouse embryos on H3K9me3 and H3K4me3 and cell lineage gene expressions in chimeric blastocysts.

**Materials and Methods:**

In our experiment, at the first step, incorporation of the GFP positive mESCs (GFP-mESCs) 129/Sv into the inner cell mass (ICM) of pre-compacted and compacted morula stage embryos was compared. At the second and third steps, H3K4me3 and H3K9me3 status as well as the expression of *Oct4, Nanog, Tead4,* and *Cdx2* genes were determined in the following groups: i. *In vitro* blastocyst derived from *in vivo* morula subjected to mESCs injection (blast/chimeric), ii. *In vivo* derived blastocyst (blast/*in vivo*), iii. *In vitro* blastocyst derived from culture of morula *in vivo* (blast/morula), and iv. *In vitro* blastocyst derived from morula *in vivo* subjected to sham injection (blast/sham).

**Results:**

Subzonal injection of GFP-mESCs at the pre-compacted embryos produced more chimeric blastocysts than compacted embryos (P<0.05). The number of trophectoderm (TE), ICM, ICM/TE and total cells in chimeric blastocysts were less than the corresponding numbers in blastocysts derived from other groups (P<0.05). In ICM and TE of chimeric blastocysts, the levels of H3K4me3 and H3K9me3 were respectively decreased and increased compared to the blastocysts of the other groups (P<0.05). Expressions of *Oct4, Nanog* and *Tead4* were decreased in chimeric blastocysts compared to the blastocysts of the other groups (P<0.05), while this was not observed for *Cdx2*.

**Conclusion:**

In the present study, embryo compaction significantly reduced the rate of incorporation of injected mESCs into the ICM. Moreover, in chimeric blastocysts, the levels of H3K9me3 and H3K4me3 were altered. In addition, the expressions of pluripotency and cell fate genes were decreased compared to blastocysts of the other groups.

## Introduction

Mouse chimeras have become a useful tool for studying the mammalian development processes, including formation of a specific cell lineage or tissue as well as gene function (1). Chimeras are the animals with two or more populations of genetically different cells or the recipient embryos with pluripotent stem cells from the same or different species (2). Previous studies showed that microinjection of embryonic stem cells (ESCs) is an efficient approach in producing good germ line- transmitted chimeras (3). It has been shown that injected ESCs into the 8-cell embryos or compacting morula can migrate into the inner cell mass (ICM) of resulting blastocysts (2, 4, 5) whose migration mechanisms has still remained to be elucidated (6).

On the other hand, chimeric animals are less viable and exhibit some abnormalities such as large offspring syndrome (LOS) and placental abruption. The abnormalities in early fetal and placental development may occur when embryos have been manipulated *in vitro* (7). Manipulation process and embryo culture condition can also change gene expression pattern and early embryo development by epigenetic factor modifications (8). Epigenetic changes, including modifications of DNA and histones without changing DNA sequence, are key regulatory factors in transcriptional activity and repression of genes in pre-implantation embryo (9). Recently the role of histone lysine methylation in embryo development has been noticed by many investigators (10). Previous studies have shown that histone H3 trimethylated at lysine 4 (H3K4me3) and histone H3 trimethylated at lysine 9 (H3K9me3) are respectively associated with active and inactive chromatin compartments (11). H3K4me3 is generally detected at the 5'-end of proximal promoters and it is one of the essential factors required for transcription activity in ICM of embryo (12). In contrast, H3K9me3 is generally localized at the promoter of repressed genes and it is required for constitutive heterochromatin formation in pericentromeric and centromeric DNA (13). Previous study has shown that de-methylation of H3K9 at the regulatory regions of ESCs significantly up-regulated Oct4 and *Nanog* gene expressions (14). 

Gamete and embryo manipulations, such as oocytes in vitro maturation (IVM), in vitro embryo production through intracytoplasmic sperm injection (ICSI) or in vitro fertilization (IVF), have negative impacts on embryo quality and epigenetic modifications (9, 15, 16). Although many studies implicate the effects of in vitro manipulation on alterations of epigenetic modification, the pattern of these alterations (including histone methylations) in chimeric embryos is still unclear. Therefore, in our study we aimed to investigate the pattern of H3K4me3 and H3K9me3 modifications in mouse chimeric blastocysts as well as the effects of this modifications on the ICM lineage specific gene expression (Oct4, Nanog) and trophectoderm (TE) gene expressions (*Tead4, Cdx2*). Moreover, effect of embryo compaction at morula stage on incorporation of the injected mESCs into the ICM as well as the effects of embryo manipulation on blastocyst quality, ICM, TE numbers and ICM/TE ratio was investigated. 

## Materials and Methods

### Animal care and chemicals

The study procedures were confirmed by the Research Ethics Committee of Avicenna Research Institute, Tehran, Iran (IR.SBMU.MSP.REC.1395.5.1). The chemical materials were obtained from Sigma-Aldrich (USA), unless otherwise mentioned in the text. The mice, C57BL/6, were procured from Pasteur institute of Iran and they were maintained in temperature- and humidity- controlled rooms at 12-hours dark/light cycles.

### Experimental groups 

**Fig.1:**
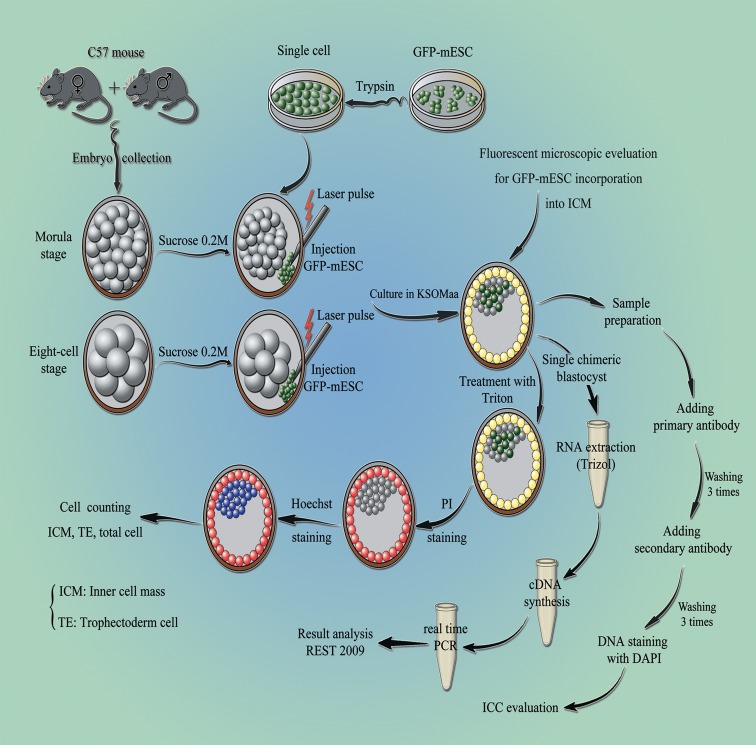
Workflow and study design for production of chimeric blastocysts. The procedure can be divided into four parts: i. Injection of GFP-mESCs, 129/Sv, into the *in vivo* derived pre-compacted and compacted mouse embryos, C57BL/6, ii. Differential staining to determine ICM and TE cells allocation of blastocysts, iii. The expression of lineage specific genes in the blastocysts derived from different approaches, using qRT-PCR, and iv. Histone methylation of H3K4me3 and H3K9me3 in blastocysts derived from different approaches using immunocytochemistry. ICM; Inner cell mass, TE; Trophectoderm, and qRT-PCR; Quantitative reverse transcription polymerase chain reaction.

This study is comprised of three experimental steps, including step 1: evaluating the effect of embryonic compaction on producing chimeric blastocyst following injection of GFP-mESCs into the subzonal space of mouse pre-compacted and compacted morula-stage embryos and step 2: determining the number of embryonic cells type, TE, ICM and total cells in blastocyst derived from different approaches including: i. *In vivo*-derived blastocyst (blastocyst/*in vivo*, control), ii. Blastocyst obtained from *in vivo*-derived morula (blastocyst/morula), iii. Blastocyst obtained from *in vivo*-derived morula which had been subjected to subzonal injection of the culture Salimi et al. medium (blastocyst/sham), and iv. Blastocyst obtained from *in vivo*-derived morula which had been subjected to subzonal mESCs injection (blastocyst/chimeric). Step 3 is composed of assessment of the some lineage specific gene expressions in ICM (*Oct4, Nanog*) and TE (*Tead4, Cdx2*), in addition to the evaluation of H3K4me3 and H3K9me3 modification in the four above-mentioned groups of blastocysts (Fig .1). 

### Embryo collection

In this study, superovulation of 8-10 weeks old C57BL/6 female mice (n=53) was performed through intraperitoneal injections of 10 IU of pregnant mare serum gonadotropin (PMSG) followed by human chorionic gonadotropin (hCG) injection after 46-48 hours. In next step, the female mice were allowed to mate with C57BL/6 male. Females with vaginal plugs were sacrificed at 2.5 days post coitum (dpc) by cervical dislocation, to collect the embryos. The embryos were cultured in KSOM, supplemented with 4 mg/ml bovine serum albumin (BSA) and amino acids (KSOMaa) under mineral oil at 37°C in a humidified atmosphere of 5% CO_2_. 

### Subzonal injection of green fluorescent protein- embryonic stem cells (GFP-ESCs) 

Mouse ESCs, 129/Sv, labelled with GFP (GFP-mESCs) were considered for subzonal injection. The cells were cultured in R2i-LIF medium consisting of 1:1 mixture of DMEM:F12 (Invitrogen Carlsbad, USA) containing 15% knockout serum replacement (KOSR), 2 mM L-glutamine, 1000 U/ml mouse leukemia inhibitory factor (LIF), 1% non-essential amino acids, 0.1 mM ß-mercaptoethanol, 100 U/ml penicillin, 100 mg/ml streptomycin, 2% ESC qualified FBS (ES-FBS), 1 µM PD0325901 and 10 µM SB431542. SB431542 and PD0325901 are two chemicals that respectively inhibit transforming growth factor b (TGF-b) and MEK signalling pathways, which are named R2i. R2i enhance ground state of pluripotency in mESCs. In the absence of mouse embryonic fibroblasts (MEFs), the mESCs grew on 0.1% gelatin-coated wells. For preparation of single cell suspension, 79% confluent mESCs were trypsinized and kept at 4°C in 1 ml of ESC medium supplemented with 0.2 m HEPES until use (5). 

The injection of GFP-mESCs in subzonal space of pre-compacted (n=42) and compacted embryos (n=36) was carried out using a Narishige micromanipulator. A number of laser beams (150 FU, Prime Tech Ltd., Japan) were applied to thin the zona pellucida (ZP) before piercing the tip of injection needle. After rinsing the inner surface of injection needle (20 µm in diameter) with 10% polyvinylpyrrolidone (PVP)-PBS, about 15 GFP-mESCs were aspirated and then injected into the perivitelline space of embryos. For expansion of perivitelline space and in order to facilitate subzonal mESCs injection, the embryos were subjected to 0.2 M sucrose medium. The GFP-mESCs-injected embryos were cultured in KSOMaa at 37°C for 24 hours in a humidified atmospheres to approach blastocyst stage (2). 

### Differential embryo staining

Differential staining of variant cell types of embryo, including TE, ICM and total cells number, was performed in each group by a previously described procedure (17). Briefly, blastocysts were permeabilized in 0.2% Triton X-100 in flushing holding medium (FHM) media for 20 seconds. They were then transferred into FHM media supplemented by 30 µg/ml propidium iodide (PI) for 60 seconds. This was followed by an incubation of blastocysts in cold ethanol supplemented by 10 µg/ml bisbenzimide (Hoechst 33342) for 15 minutes and immediately mounted on glass slides using glycerol. Finally, the stained blastocysts were observed and counted using an epifluorescent microscope (IX71 Olympus, Japan). In this study, 15 blastocysts were considered for each group. 

### Immunofluorescence staining of H3K9me3 and H3K4m3 

In each group, the ZP of blastocysts was completely dissolved by incubating them with acidic Tyrode (pH=2.5) for 30 seconds. The embryos were washed three times by phosphate-buffered saline (PBS) added to 0.1% polyvinyl alcohol (PVA) and 0.1% Tween-20. They were then fixed in 4% paraformaldehyde (pH=7.4) for 30 minutes. Subsequently, the fixed embryos were treated by 0.3% Triton X-100 for one hour in PBS. For blocking, these blastocysts were kept in PBS, followed by adding 2% bovine serum albumin (BSA) to them for 40 minutes at 25°C. They were next treated with primary anti-H3K4me3 (1:200, Abcam, USA) antibody for one hour at 25°C and anti-H3K9me3 (1:200, Abcam, USA) antibody overnight at 4°C. The embryos were then washed three times (10 minutes each) with 0.1% PVA+0.1% Tween-20 diluted in PBS, and they were then treated with the secondary antibody, goat IgG anti-mouse (PE/Cy5.5, 1:500, Abcam, USA) in blocking solution for 60 minutes at 37°C, according to the manufacturer’s instructions. After washing with PBS containing 0.1% PVA+0.1% Tween-20, for 10 minutes, DNA was stained for 10 minutes with 15 µg/ml 6-diamidino-2-phenylindole (DAPI, Thermo Fisher Scientific, USA). The samples were then mounted on the slides by glycerol. Each experiment was biologically replicated three times and at least 20 blastocysts were evaluated in each group. In each experiment, embryos without primary antibody were stained, as negative controls. The slides were evaluated using an automated epifluorescent microscope (Nikon, Japan). The fluorescence intensity of blastocyst images was evaluated using ImageJ software (NIH Image, USA). 

### RNA isolation

Total RNA was isolated from single blastocyst using Trizol reagent (Life Technologies, Belgium) according to the manufacture’s instruction. Briefly, to homogenate the samples, 50 µl Trizol and 25 µl chloroform were added to each sample. After precipitating with isopropanol, RNA was washed with 70% ethanol and total RNA was diluted in 10 µl RNase-free water. Total RNA was then kept at -80°C. In our study, five blastocysts were considered in each group. 

Before cDNA synthesis, the purity and concentration of isolated RNA was measured using a spectrophotometer (Picodrop Real-Life, UK). cDNA was produced using Prime Script QuantiTect Kit (Qiagen, Germany). Reactions were carried out in RNase-free tubes in a total volume of 20 µl, containing 2 µl gDNA, 6 µl total RNA, 4 µl RT buffer, 1 µl enzyme and 7 µl RNase-free water at the following condition: 42°C for 2 minutes, 42°C for 15 minutes and 95°C for 3 minutes. For long term storage, cDNA were kept at -20°C. 

### Quantitative reverse transcription polymerase chain reaction

In this study, quantitative reverse transcription polymerase chain reaction (qRT-PCR) was used to assess the expression of: 

Oct4

F: 5´-CGTGTGAGGTGGAGTCTGGA-3´,

R: 5´-GCTGATTGGCGATGTGAGTG-3´,

Nanog-

F: 5´-CTGAGGAGGAGGAGAACAAGGTC-3´,

R: 5´-CATCTGCTGGAGGCTGAGGTA-3´,

Tead4

F: 5´-CGGAGGAAGGCAAGATGTATG-3´, 

R: 5´-ACCTGGATGTGGCTGGAGAC-3´and 

Cdx2

F: 5´-GCTGCTGTAGGCGGAATGTAT-3´, 

R: 5´-CTCCCGACTTCCCTTCACC-3´ 

using Rotor Gene Q instrument (Qiagen, Germany). qRT- PCR reactions were done in a final volume of 10 µl including 5 µl SYBR green (Takara, Japan), 0.2 µl of each forward and reverse primers (10 µM), 2 µl cDNA template (ten-fold diluted), and 2.6 µl nuclease free water. The thermal cycling was performed in 2 steps with following condition: one cycle of 95°C for 30 seconds (holding time), followed by 50 cycles of 95°C for 5 seconds and 60°C for 30 seconds. High- resolution melting curve analysis was performed in a ramp rate of 0.2°C from 72°C up to 95°C. 

Gapdh-

F: 5´-TTCCAGTATGATTCCACCCAC-3´,

R: 5´-ACTCAGCACCAGCATCACC-3´ and 

H2afz

F: 5´-CTCGTCTCTTCCTCGCTCGT-3´,

R: 5´-CGTCCGTGGCTGGTTGTC-3´ 

were considered as internal endogenous housekeeping genes. At least, three replications from each cDNA sample were evaluated and the expression level of the gene was normalized against *H2afz* and *Gapdh*. Relative expression of the genes was determined by REST 2009 Software (Qiagen, Germany).The expression levels were reported as mean ± standard deviation (SD), while significant difference was reported as P<0.05.

### Statistical analysis

The rates of development to the blastocyst in pre-compacted and compacted embryos following mESCs injection and the incorporation of injected mESCs into ICM were analysed by non-parametric analysis test (Mann Whitney) and expressed as mean ± standard error of the mean (SEM). The blastocyst cell number and fluorescent intensity of histone methylation were evaluated with one-way ANOVA post hoc tests and expressed as mean ± SD. Analyses were conducted using SPSS statistical program (SPSS Inc., USA). Comparisons were considered statistically different, if the p-value was less than 0.05. Gene expression of the each groups were evaluated by one-way ANOVA, REST 2009 Software (Qiagen, Germany). 

## Results

### Generation of mouse chimeras

Integration of subzonal injected GFP-mESCs into the ICM of resulting blastocysts were significantly higher in pre-compacted (31/42) than compacted (13/36) morulastage embryos (P=0.012, Table 1). As shown, the injected GFP-mESCs were incorporated into the ICM of resulting chimeric blastocysts using epifluorescent microscope. There was no difference in the blastocyst rate and developmental block between morula-stage embryos receiving mESCs at pre-compacted or compacted stages (Table 1). 

### Blastocyst cell count

**Fig.2:**
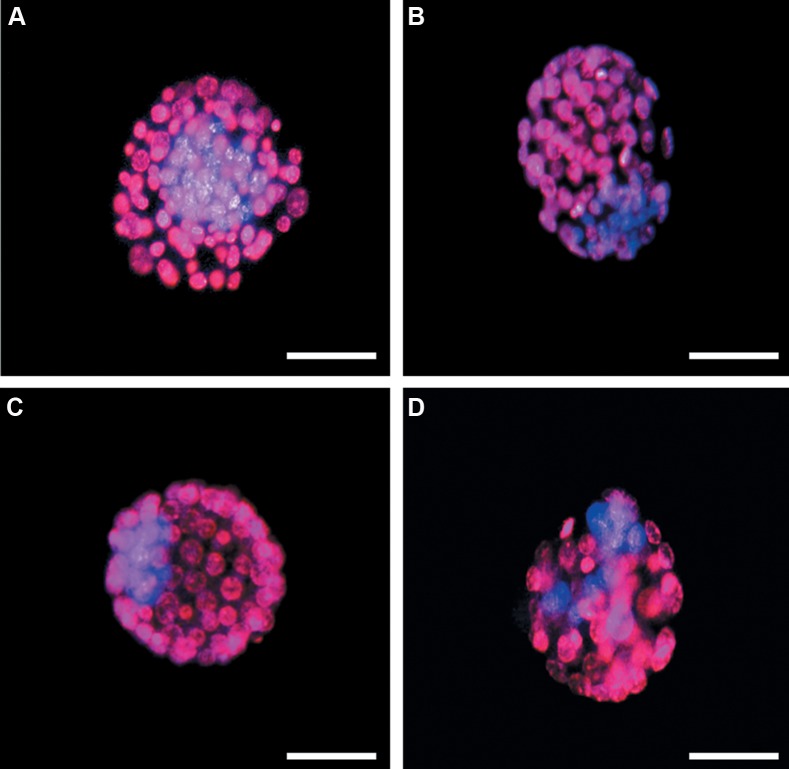
Epifluorescent microscopic imaging of mouse chimeric blastocysts produced by different approaches. ICM and TE nuclei were respectively stained with Hoechst 33342 (blue) and PI (red). **A.** Blastocyst/*in vivo*, **B.** Blastocyst/morula, **C.** Blastocyst/sham, and **D.** Blastocyst/ESCs injecton (scale bar: 50 µm). ICM; Inner cell mass, TE; Trophectoderm, and PI; Propidium iodide.

As it has been shown in Table 2, the number of variant cell types of embryo, including total cell numbers, TE and ICM was measured in four groups to determine the blastocyst quality (Fig .2). Chimeric blastocysts had significantly fewer total cell, ICM and TE cell numbers compared to the other groups (P<0.05). The average number of ICM was respectively 9.7 ± 1.4 and 19.75 ± 1.3 in chimeric and derived blastocysts *in vivo*. The ration of ICM to TE cells (ICM/TE) was decreased in chimeric blastocysts compared to blastocyst/*in vivo* (P<0.05). 

**Table 1:** Subzonal injection of mESCs in morula stage of pre-compacted as well as compacted mouse embryos, and incorporation of mESCs into ICM of resulting blastocysts


Injected morula	Produced blastocyst	Blastocyst		Blocked embryo
		Incorporated mESCs	Non-incorporated mESCs	

Compacted (n=36)	34 (94.4 ± 4.8)	13 (36.1 ± 7.5) ^a^	23 (63.9 ± 10.6 )^a^	2 (5.5 ± 2.8)
Pre-compacted (n=42)	40 (95.2 ± 2)	31(73.8 ± 4.5 )^b^	11 (26.2 ± 3.1)^b^	2 (4.8 ± 1)
Total number: (n=78)	74 (94.67 ± 2.5)	44 (53.77 ± 7.17)	34 (40.90 ± 7.38)	4 (5.317 ± 1.56)


Data are presented as n (% ± SEM). ^a, b^; The numbers with different uppercase letters at the same column are significantly different (P<0.05), mESC; Mouse embryonic stem cells, and ICM; Inner cell mass.

**Table 2:** The number of variant cell types of blastocysts obtained from different approaches


Groups	Number of ICM cells	Number of TE cells	Total cell number	ICM:TE
				Ratio

Blastocyst/*in vivo*	19.75 ± 1.3^a^	64.5 ± 14.1^a^	84.25 ± 17^a^	0.31 ± 0.09^a^
Blastocyst/morula	16.57 ± 1.5^b^	55.28 ± 8.9^a^^,^^b^	71.85 ± 9.5^a^^,^^b^	0.30 ± 0.1^a^
Blastocyst/sham	12.83 ± 0.8^c^	48.5 ± 6.5^b^^,^^c^	61.33 ± 8.5^b^	0.26 ± 0.12^b^^,^^c^
Blastocyst/ESCs injection	9.7 ± 1.4^d^	38 ± 9^c^	47.7 ± 9.6^d^	0.25 ± 0.15^c^


Data are presented as mean ± SD. ^a-d^ ; The numbers with different uppercase letters at the same column differ significantly (P<0.05), TE; Trophectoderm, ICM; Inner cell mass, and EScs; Embryonic stem cells.

### Immunocytochemistry analysis of H3K9me3 and H3K4me3

**Fig.3:**
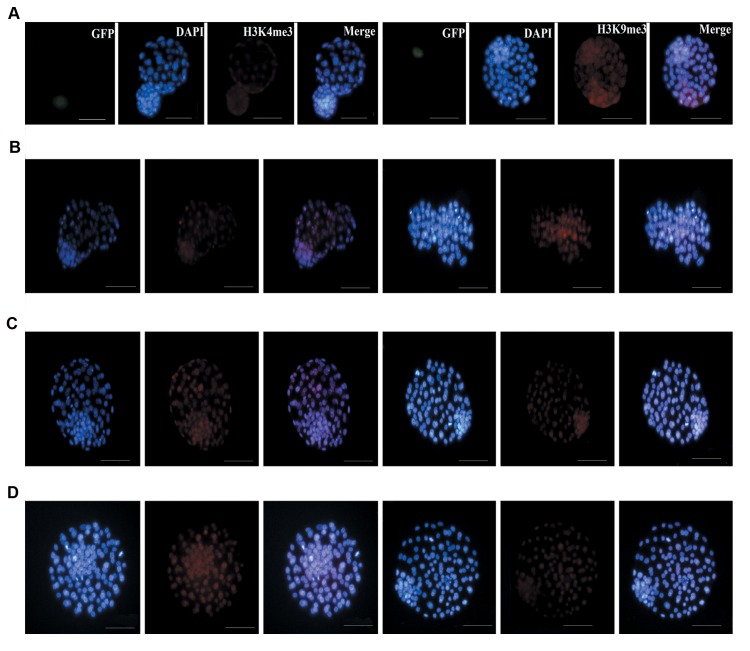
Immunostaining. Immunocytochemistry staining of H3K4me3 (left) and H3K9me3 (right) in chimeric blastocysts and blastocysts derived from the other groups: **A.** Blastocyst/embryonic stem cells (ESCs) injection, **B.** Blastocyst/sham, **C.** Blastocyst/morula, and **D.** Blastocyst/*in vivo*; control. The nuclei (blue) were stained with DAPI. The H3K4me3 and H3K9me3 were stained with anti-Mouse IgG (red). The merged images of H3K4me3 and H3K9me3 with DNA are purple (scale bars: 50 µm).

**Fig.4:**
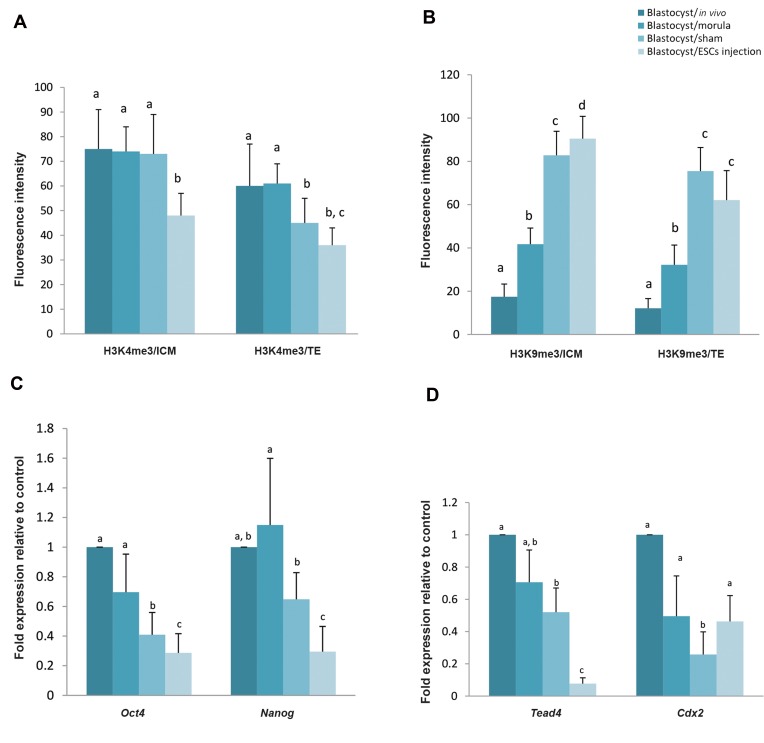
Three methylation of H3K4 and H3K9 as well as gene expressions in blastocysts produced by different approaches. **A.** H3K4me3 expression, **B.** H3K9me3 expression, **C.** Expression of the ICM genes, and **D.** Expression of the TE genes in mouse blastocysts produced by different approaches. a, b, c the columns with different type of the lowercase letters are significantly different (P<0.05). Data are shown as mean ± SD. ICM; Inner cell mass and TE; Trophectoderm.

Methylation of H3K4 and H3K9 in the TE and ICM cells of blastocyst were measured by immunocytochemistry assay (Fig .3). As shown in Figure 4A, methylation of H3K4 in the ICM and TE of chimeric blastocysts was decreased in comparison with the other groups (P<0.05). Concerning tri-methylation of H3K9 in ICM, the highest rate was observed in chimeric embryos. Tri-methylation of H3K9 in ICM and TE was significantly higher in chimeric and sham groups compared to the other groups (P<0.05). On the other hand, there was no significant difference between expression of H3K9me3 in TE of sham groups and chimeric groups (P. 0.05, Fig .4B). 

### Inner cell mass and trophectoderm gene expressions 

Relative expression analysis of particular lineage specific genes in the ICM and TE cells represented some differences. Oct4, Nanog and Tead4 relative expressions in chimeric blastocysts was significantly lower than blastocysts in sham and control groups (P<0.05). However, no significant difference was observed for *Cdx2* between chimeric blastocysts and those derived from the other groups, except the sham group. Indeed, *Cdx2* expression in blastocysts derived from sham group was significantly lower than the other groups (Fig .4C, D, P<0.05). 

## Discussion

Currently, mESCs microinjection is a highly stable and reproducible technique which can produce full germ line- transmitted chimeras (1). In the present study, integration of mESCs into the ICM in pre-compacted embryos was significantly higher than compacted type. Our results was in agreement to the reports of Tokunaga and Tsunoda (18) indicating that mESCs injection into the 8-cell embryos, 2.5 dpc before formation of ICM, led to the higher incorporation of injected cells into the ICM. In our study, blastocysts were incubated in 0.2 M sucrose medium prior to mESCs injection into the subzonal cavity of morulastage embryos. Our results were consistent with previous studies clearly showing that hypertonic microinjection method can generate the embryos with high percentages of chimerism. The precise cellular mechanism underlying this phenomenon is not yet clear (3, 19). 

In our study, the number of variant cell types of embryo including total cells number, ICM and TE, as well as the ICM/TE ratio, was reduced in chimeric blastocysts compared to blastocysts *in vivo*. It seems that in vitro manipulation of the embryo can compromise the quality of produced blastocysts. So that, the number of ICM cells in the injected mESCs group was lower than that of the other groups. In other words, the number of ICM cells was reduced by increasing embryo manipulation. As expected, reducing the variant cell number of embryo, TE and ICM, as well as the abnormal changes in blastocyst cells allocation, reduced the quality and post implantation development of blastocyst. It has also been shown that the rate of embryonic cell proliferation and the ICM/TE ratio in the blastocyst leads to placental abnormalities and LOS (20). In the cloned embryos with a small number of variant cell types, functional role of TE cells for successful implantation was reduced (21). 

It has been demonstrated that mESCs-derived chimeras suffer from reduced viability and other anomalies such as altered growth rate and body weight (22). These abnormalities could be mainly due to the changes in gene expression of TE and ICM cells, causing by embryo manipulation and in vitro culture (23, 24). In this context, epigenetic modification has a profound effect on gene expression. Histone modification plays an important role in transcription activity via methylation of lysine and chromatin structure remodelling in pre-implanted embryo (25). Embryo manipulations, such as SCNT, ICSI and cryopreservation, modify somewhat the normal pattern of H3K9me3 and H3K4me3 methylation (16, 17, 26). However, there is no study to assess methylation of H3K4 and H3K9 in chimeric embryos and evaluate the role of this modification on gene expression.

It has been shown that H3K4me3 is enriched in transcription starting site of some transcription factors, including *Nanog, Oct4* and *Sox2* genes, which have regulatory role in gene expression (27). *Oct4* is one of the main genes, known to act as a master regulator of pluripotency (28). It belongs to POU family of transcription factor genes. It is found in the promoter and enhancer regions of many genes. *Oct4* also regulates expression of *Nanog, Sox2* and other genes modulating the cell fate during early embryo development (29, 30). In our study, H3K4me3 in ICM cells of chimeric blastocysts was decreased, in comparison with the other groups. It probably reduced the expression of certain specific genes, including Oct4 and Sox2 in ICM. This was in agreement with many studies indicating that *in vitro* derived embryos and embryo manipulation can alter methylation pattern, consequently leading to the change in expression of pluripotency genes, compared to the *in vivo* derived type (28, 31). 

Our study shows that in chimeric embryos, duration and severity of the manipulation *in vitro* were more than the other groups. Based on that and in accordance with the previous studies (31, 32), duration and severity of the embryo manipulation may lead to more epigenetic alterations in H3K9me3 and H3K4me3, consequently reducing the ICM and TE gene expressions (32-34). According to the our results, because of difference in the type and duration of manipulation *in vitro*, there was a significant trend in reduction of the ICM cells H3K4me3 methylation, but not TE cells. Another possibility for decreasing H3K4 methylation in ICM cells of chimeric blastocysts, compared to the other groups, might be due to the possible interactions between injected mESC and ICM cells in chimeric blastocysts. Methylation of H3K4 in chimeric blastocysts was also reduced compared to other groups, although the difference between sham and chimeric groups was not significant. 

In the normal process of blastocyst development, generating ICM and TE cells, there is a relationship between *Oct4* and *Cdx2* expressions. As such, the increase in *Oct4* expression leads to the reduction of *Cdx2* expression in ICM cells and vice versa in TE cells. *Cdx2* as a transcription factor is responsible for embryo compaction and TE lineage formation (35). In our study, despite the significant decrease in expression of *Oct4, Nanog* and *Tead4*, down-regulation of *Cdx2* expression was not significant in chimeric blastocysts under the impact of manipulation compared to *in vivo*-derived counterparts. Now, the question is why despite the reduced expression of other genes, expression of *Cdx2* has not been declined? Whether genes associated with cellular fate (Tead4) and pluripotency (*Oct4* and *Nanog*) are more sensitive than TE gene (*Cdx2*) in the face of inappropriate culture conditions or manipulation? However, there are studies indicating no significant difference in *Cdx2* expression between embryos with (cloned and ICSI embryos) and without manipulation (36). 

H3K4me3 is generally associated with active chromatin, whereas H3K9me3 preferentially correlates with heterochromatin and transcription repression (37). Here, we found a converse relationship between H3K9me3 and gene expression in ICM and TE of chimeric embryos. In other words, increasing level of H3K9me3 leads to the reduction of *Nanog, Oct4* and *Tead4* gene expressions in chimeric embryos, compared to *in vivo *derived blastocysts. It is hypothesized that reduction of H3K9me3 level by optimizing culture condition can improve epigenetic pattern in the chimeric embryos. Consistently, it has been shown in mouse and porcine cloned embryos (38, 39). In agreement with our study, H3K9me3 could down- regulate the pluripotency gene expressions in the cloned embryos (38). Regarding the *Cdx2* expression, despite decreasing trend of gene expression in the first three groups, the expression in test group (chimeric blstocysts) was increased in comparison with the sham group. One explanation for the significant decrease in *Cdx2* expression in the sham, compared to test group, might be due to the higher, though insignificant, level of H3K9me3 in sham group. Alder et al. (40) indicated that down-regulation of H3K9me3 in TE leads to the activation of *Cdx2* transcription. As expected, in our study, H3K9 histone methylation of the ICM cells was significantly increased in chimeric blastocysts compared to the sham group. It was in accordance to the general principle that further manipulation will cause more epigenetic alterations. In other words, more invasive operations, as with chimeric embryos, cause more epigenetic changes. 

Concerning the level of H3K9me3 in TE cells, despite determining consistently increased level of methylation contrary to our expectations, there was no significant difference between the chimeric and sham groups. There are, however, other factors that can alter gene expressions, such as DNA methylation and histone modification, which may affect the gene expression pattern in chimeric embryos, compared to the other groups. Apart from *Cdx2*, the expression of *Tead4* in TE cells was decreased, while it was increased in the embryo manipulation. Thus, the minimum expression level was shown in chimeric blastocysts, compared to the other groups. These alterations followed the pattern of H3K9me3 changes in TE cells. 

## Conclusion

In our study, the embryonic stage had a profound effect on production of chimeric blastocyst. Thus, embryo compaction significantly reduced the rate of mESCs incorporation to the ICM. Moreover, alterations in the levels of H3K9me3 and H3K4me3 could reduce the pluripotency and cell fate gene expressions, due to embryo in vitro culture and its manipulation.
